# Episodic records of jellyfish ingestion of plastic items reveal a novel pathway for trophic transference of marine litter

**DOI:** 10.1038/s41598-018-24427-7

**Published:** 2018-04-17

**Authors:** A. Macali, A. Semenov, V. Venuti, V. Crupi, F. D’Amico, B. Rossi, I. Corsi, E. Bergami

**Affiliations:** 1 Department of Ecological and Biological Sciences, Ichthyogenic Experimental Marine Centre (CISMAR), Borgo Le Saline, Tuscia University, Tarquinia, 01016 VT Italy; 2 N. A. Pertsov White Sea Biological Reseach Station, Department of Biology, Lomonosov Moscow State University, 1-12, Leninskie Gory, Moscow, 119234 Russia; 3 Department of Mathematical and Computer Sciences, Physical Sciences and Earth Sciences, University of Messina, Viale Ferdinando Stagno D’Alcontres 31, Messina, 98166 Italy; 4 Elettra Sincrotrone Trieste S.C.p.A., S.S. 14 Km 163.5 in Area Science Park, Trieste, I-34149 Italy; 5 Department of Physical, Earth and Environmental Sciences, University of Siena, Via Mattioli 4, Siena, 53100 Italy

## Abstract

Invertebrates represent the most plentiful component of marine biodiversity. To date, only few species have been documented for marine litter intake. Here, we report for the first time the presence of macroplastic debris in a jellyfish species. Such novel target to plastic pollution highlights an under studied vector of marine litter along marine trophic web, raising further concern over the impact on marine wildlife.

## Introduction

The number of field studies showing the impacts of anthropogenic litter on marine organisms is increasing. To date, plastic ingestion has been documented for 233 marine vertebrates^1^. However, despite invertebrates representing the primary component of marine biodiversity, litter ingestion has been reported in only a few species^2–4^. Plastic ingestion is associated with both physical damages such as gut blockage, reduced energy reserves and starvation^1^, and potential toxicity due to persistent, bioaccumulative and toxic (PBT) substances adsorbed onto the plastic surface^5–7^ or those leached from the polymer matrix, such as phthalates and flame retardants, known also as endocrine disruptors^8–11^. Thus, plastic debris provides a pathway for these chemicals to enter marine ecosystems through plastic ingestion^3,10,12^.

Here we present the first evidence of marine litter internalised by the mauve stinger *Pelagia noctiluca* (Forsskål, 1775), the most abundant jellyfish species in the Mediterranean Sea^13^.

As with the distribution of plastic debris, the dispersal of this species mainly depends on the combined effect of local winds and currents^14–16^, concentrating this organism in regions with a high concentration of floating litter. *P. noctiluca* is considered an opportunistic predator, feeding on zooplankton of a broad size and taxonomic range, as a result of an adaptive strategy in the open ocean where potential prey are highly diverse^17^. The mauve stinger also represents a key food source for pelagic top predators. The jellyvorous guild in the Mediterranean includes two specialists (ocean sunfish and loggerhead sea turtle) and several opportunist feeders (Bluefin tuna, little tunny, spearfish, swordfish and blue butterfish), most of which are of commercial interest^18,19^.

Furthermore, medusae are known to supply an important contribution of carbon into pelagic and deep seafloor communities after blooms senescence^20^.

Hence, any impact of marine litter on this species might have consequences on its predators as well as on the marine primary productivity at an ecosystem scale.

## Results

**Figure 1:**
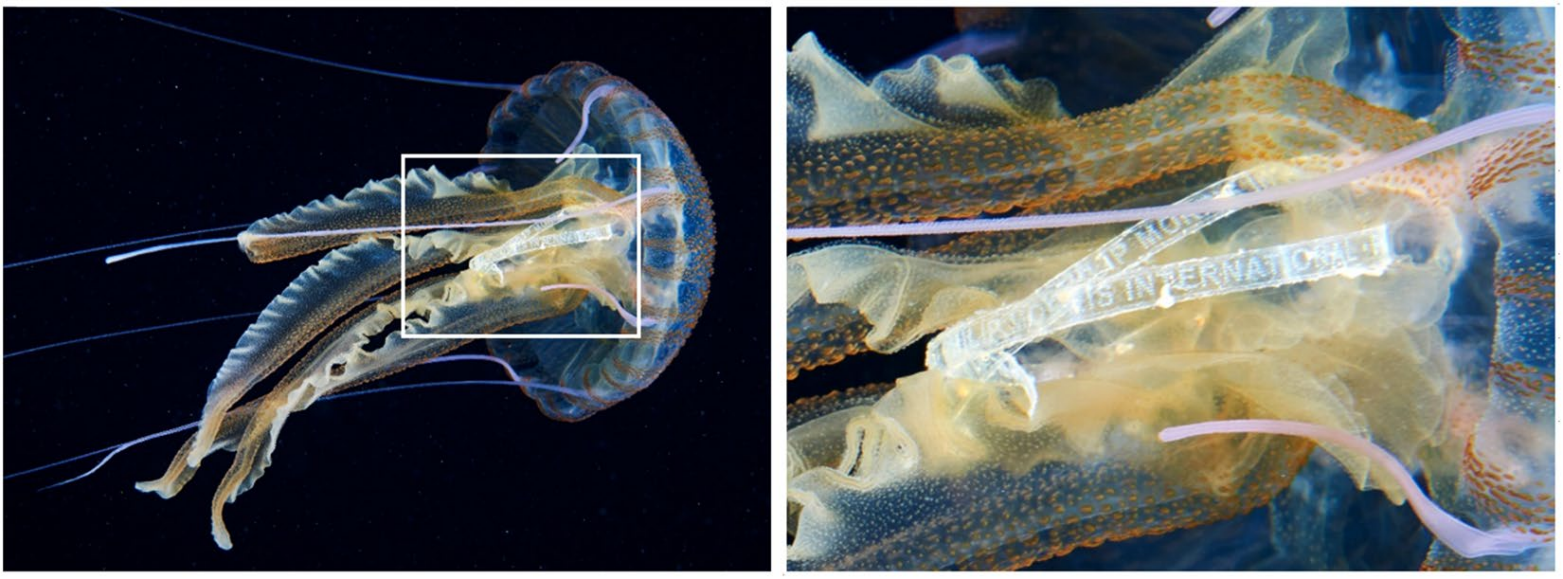
Interaction between plastic debris and jellyfish. High resolution images showing a swimming mauve stinger *P. noctiluca* observed in the field (left) with a plastic lace of a famous cigarette brand among the oral lobes (white square, enlarged on the right).

During the Mediterranean campaign of the “AQUATILIS EXPEDITION”^21^ on Ponza Island (Tyrrhenian Sea), specimens of *P. noctiluca* were found along with floating anthropogenic litter of different size, colour, shape and type. Some of these materials were found trapped among the oral lobes of the jellyfish (Fig. 1) or retained inside their hood (Fig. S1). To the best of our knowledge, no similar observations have been reported so far for jellyfish, although their blooms have been observed in regions of plastic accumulation^22^.

**Figure 2:**
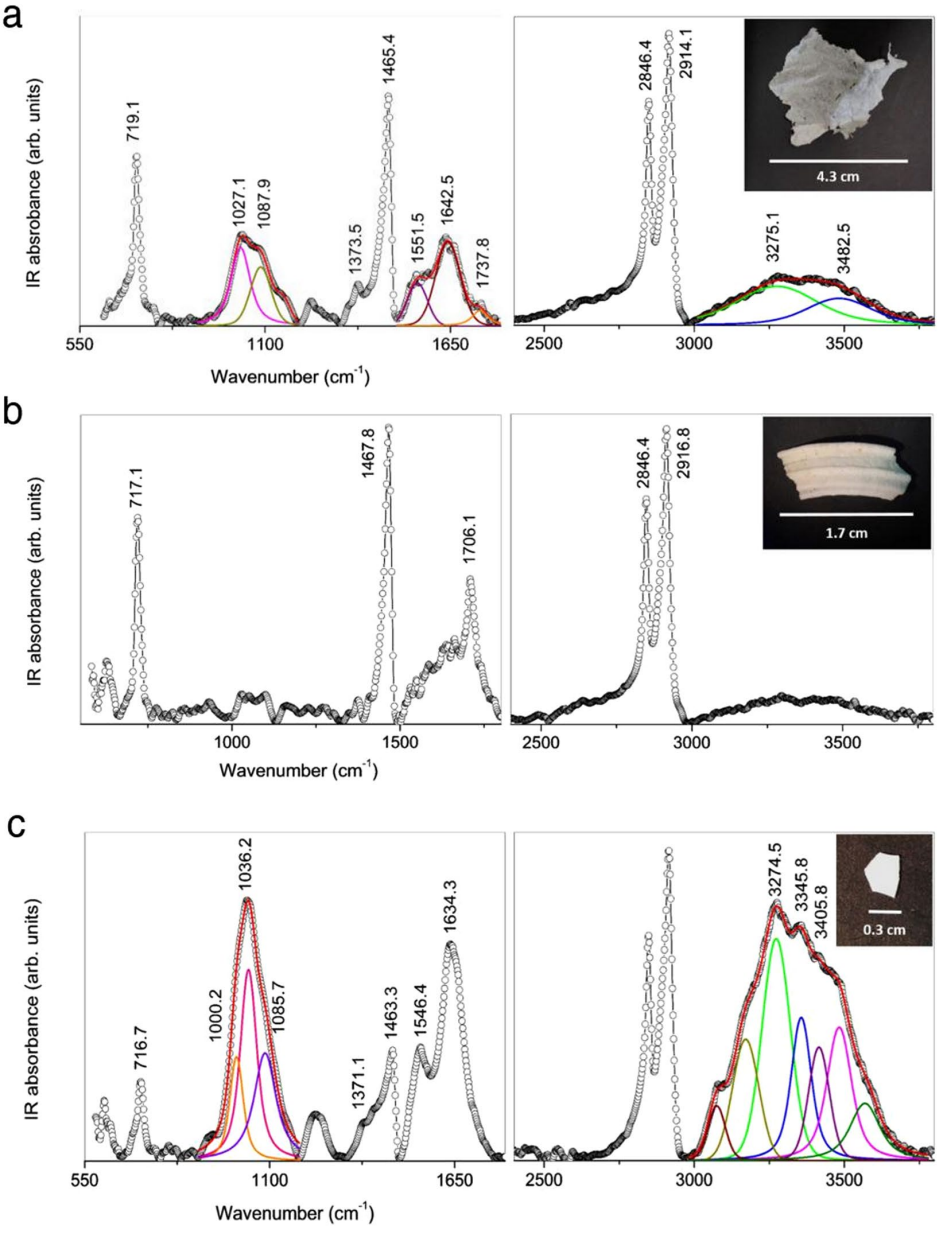
Characterization of marine litter internalised by jellyfish. ATR-FTIR spectra of the marine debris (shown in the square on the right) extracted from specimens of the mauve stinger *P. noctiluca* from Tyrrhenian Sea, classified as HD-PE sheet (**a**), PE fragment (**b**) and metal-based paint fragment (**c**).

Although only a small sample size was investigated, field observations showed a strong interaction between *P. noctiluca* and drifting litter. In order to determine the effective ingestion of anthropogenic litter, a total of 20 specimens of *P. noctiluca* were collected using hand nets. Four individuals presented plastic debris inside the gastrovascular cavity. The characterization of such litter through ATR-FTIR and Raman spectroscopy revealed two macro-sized plastics (>1 cm) constituted by high-density polyethylene (HD-PE) (Figs 2a, S2A), a flame retardant polyethylene (PE) (Figs 2b, S2B) and a zinc-rich paint fragment (Fig. 2c) (see also Supporting Information).

## Discussion

We hypothesize that the presence of marine litter in the gastrovascular cavity of *P. noctiluca* specimens (Fig. 2) was due to active ingestion of such fragments wrongly recognised as food. Marine organisms have been reported to ingest plastics as a result of visual^23–26^ or tactile^27^ misidentification, or attracted by flavouring organic compounds on the plastic surface^23,28^.

In cnidarians, the discharge mechanism of cnidocytes is evolutionarily conserved among different classes^29^. Prey capture and feeding consist in a sequence of chemically-mediated behaviours: (a) discharge of cnidocytes by compounds usually associated with cell membranes, mucin and chitin of the prey, such as N-acetylated sugars; (b) retraction of tentacles triggered by endogenous compounds to move captured prey to the mouth; (c) ingestion of the prey, promoted by a reversed ciliary beating on the mouth and pharynx^30,31^.

Due to its specific chemical and physical properties, drifting weathered litter may be mistaken by nematocysts as food. PE (considering both low- and high-density) is one of the main polymers produced worldwide^32^ and accounts for the majority (52%) of the marine litter found in Mediterranean surface waters^33^. Given its specific gravity, (ranging from 0.91 to 0.94^34^), it usually floats near the sea surface, closely behaving as neuston organisms, thus potentially inducing a meccano- and chemoreceptors reaction of cnidarians, which mistake it as prey. Laboratory experiments conducted on hard corals^35^ showed nematocyst discharge and ingestion of different microplastics (including PE), suggesting that the presence of phagostimulants potentially related to toxic compounds found in the polymer matrix are promoting chemoreception. Other marine pollutants also induce feeding behaviour in Cnidaria^36,37^. For instance, heavy metals, which can be found adsorbed to plastic debris in the marine environment^6,7^, have been shown to affect the chemoreceptors in *P. noctiluca*^38^.

The mauve stinger can consume prey equivalent to >50% of its body weight (wet weight) per day^17^, with the ability to ingest gelatinous prey of considerable size compared to its bell dimension^39^. This aspect further supports our hypothesis of an active ingestion of large anthropogenic fragments in *P. noctiluca*.

Studies on corals showed a retention rate of 5.7% of microplastics in mesenterial tissue within the gut cavity after 24 h of recovery^35,40]^. This is surprising, since anthozoans can digest food rapidly retaining non-food particles for an average of 50 minutes^41–43^. The residence time of plastics could be related to the polymer, size and shape, and further studies are needed to elucidate the fate of the ingested plastics in Cnidaria.

The biomass of gelatinous zooplankton in the epipelagic region of the Mediterranean usually ranges between 1–10 kg 100 m^−3^, with the biomass of *P. noctiluca* reaching values up to 24 kg 100 m^−3 ^^44^, making it a valuable food source for several pelagic organisms^18,19,45^. Studies using stable isotopes suggest that gelatinous zooplankton, such as the mauve stinger represent between 30 and 60% of the diet of the Bluefin tuna^19^. Therefore, an important amount of marine litter may be transferred through jellyfish to pelagic predators^19^ suggest that fish, such as tuna, are unable to consume the required biomass of jellyplankton in a single meal, thus continuous consumption of gelatinous plankton and associated plastic debris could be possible.

Our findings highlight the vulnerability of medusae to plastic pollution found suspended in the water column and transported through currents. The ability of jellyfish, and specifically *P. noctiluca*, to internalise low-density macroplastic and other anthropogenic debris leads to a reinterpretation of the impact of marine litter on their common predators, previously thought only to mistake floating plastics as prey.

Here, we hypothesise that jellyfish could act as vector of plastics along marine trophic webs, raising further concern on the impact of plastics on marine organisms. Further studies are urgently needed to understand any potential effect for this species and the real extent of trophic transfer of plastic debris.

## Methods

This study was carried on within the “AQUATILIS EXPEDITION”^21^ on the Mediterranean Sea around Ponza Island (Tyrrhenian Sea, Italy) in September 2016.

Due to its bathymetric profile, the site is particularly exposed to prominent surface and upwelling streams. This complex hydrodynamism likely concentrates planktonic species and floating litter in the same geographic area.

Mauve stinger specimens observed during the night diving campaign clearly contained anthropogenic material trapped among oral lobes and retained in the gastrovascular cavity (Figs 1, S1). Of the specimens collected on board (N = 20; average bell size = 4.7 cm), three large plastic debris were extracted from the gastrovascular cavity and one piece of polystyrene foam colonized by barnacles was detached from the oral lobes. All fragments were further analysed through Attenuated total reflection Fourier-transform infrared (ATR-FTIR) and UV Resonant Raman spectroscopies. ATR-FTIR spectra were collected at room temperature by a Bomem DA8 FTIR spectrometer using a thermoelectrically cooled deuterated triglycene sulphate (DTGS) detector in combination with a KBr beam splitter and a Globar source. Fragments without any *a priori* treatment were placed in a Golden Gate ATR system based on ATR technique. Spectra were recorded in the 500 ÷ 4000 cm^−1^ region. Each spectrum was collected in a dry atmosphere in order to avoid unnecessary contributions from air humidity, with a resolution of 4 cm^−1^ and an average of 100 repetitive scans to ensure high reproducibility and good signal-to-noise ratio. All spectra were normalized for taking into account the effective number of absorbers, no mathematical corrections were applied (e.g. smoothing), and all spectroscopic manipulations such as subtraction of a baseline were performed using Spectra Calc GRAMS software (Galactic Industries, Salem, NH, USA). When necessary, the experimental spectrum bandwidth was performed by curve fitting into Voigt profiles, using the routine provided by the PeakFit 4.0 software package. The statistical parameters defined in the software manual were used as a guide for the “best-fit” and were released during the iterative procedure until convergence was achieved. “Best-fit” has been defined as r^2^ ∼ 0.99999 for all investigated samples. For the recognition of polymer, the KnowItAll IR Spectral Library (Bio-Rad Laboratories Inc., USA) was used, resulting in a score of 93.2% (Fig. 2a), 94.10% (Fig. 2b) and 87.56% (Fig. 2c).

UV Resonant Raman measurements were carried out at the IUVS beamline at Elettra synchrotron Radiation facility (Trieste, Italy). An excitation source of 266 nm with beam-power of 1 mW was employed. The scattered light was collected by using a backscattering geometry configuration. A single Czery-turner spectrometer (focal distance of 750 mm, equipped with an 1800 g/mm holographic grating) coupled with a Peltier-cooled back-thinned CCD was used to acquire the final Raman spectra,with a spectral resolution of 5 cm^−1^.

### Data availability

No datasets were generated or analysed during the current study.

## Electronic supplementary material

Supplementary Information
