# Supplementary material for: Episodic records of jellyfish ingestion of plastic items reveal a novel pathway for trophic transference of marine litter
Source: Sci Rep. 2018 Apr 17;8:6105. doi: 10.1038/s41598-018-24427-7 (PMC5904158; doi:10.1038/s41598-018-24427-7)
Supplement: Supplementary file 1 — Supplementary Information [file 41598_2018_24427_MOESM1_ESM.docx]

**Episodic records of jellyfish ingestion of plastic items reveal a novel pathway for trophic transference of marine litter**

Macali A.**^A^**, Semenov A.**^B^**, Venuti V.^C^, Crupi V. **^C^**, D’Amico F.**^D^**, Rossi B.**^D^**, Corsi I.**^E^**& Bergami E.**^E^**

**^A^** Tuscia University, Department of Ecological and Biological Sciences, Ichthyogenic Experimental Marine Centre (CISMAR), Borgo Le Saline, 01016 Tarquinia, VT, Italy

**^B^** N. A. Pertsov White Sea Biological Reseach Station, Lomonosov Moscow State University, Department of Invertebrates Zoology, 1-12, Leninskie Gory, Moscow 119234, Russia

**^C^** University of Messina, Department of Mathematical and Computer Sciences, Physical Sciences and Earth Sciences, Viale Ferdinando Stagno D’Alcontres 31, 98166 Messina, Italy

**^D^** Elettra Sincrotrone Trieste S.C.p.A., S.S. 14 Km 163.5 in Area Science Park, I-34149 Trieste, Italy

**^E^** University of Siena, Department of Physical, Earth and Environmental Sciences, Via Mattioli 4, 53100, Siena, Italy

**Supporting Information**

These are three pages of supporting information, containing a total of 2 figures, associated to this manuscript.

**Supporting Information**

According to the definition proposed by ^[1]^, macro-sized plastics (> 1 cm) and a zinc-rich paint fragment were identified through by Attenuated Total Reflection Fourier-Transform Infrared (ATR-FTIR)(Fig. 2) and further by UV-Raman spectroscopy (Fig. S2). Spectra shown in Fig. 2A refer to a macroplastic of 4.3 cm constituted by high density polyethylene (74%), xanthan gum (15%) and fatty acid (11%). The macroplastic in Fig. 2B (1.7 cm total length) resulted less heterogeneous, with 85% of flame retardant polyethylene and 15% of coextrudable adhesive resin. The latter (Fig. 2C) resulted in a paint fragment of 0.3 cm total length, characterised by a fatty amide derivative (42%), triethanolamine (37%) and an inorganic white zinc phosphate pigment (23%).

UV-Raman spectra of plastic sample 1 (corresponding to the fragment shown in Fig. 2A) matches with the HD-PE spectra available in the literature ^[2]^, with further characteristic regions between 1500 and 1700 cm^-1^ not directly comparable with ^[2]^, which are derived from the other components present, as identified by ATR-FTIR. Conversely the UV Raman spectra collected on Plastic sample 3 (corresponding to the fragment shown in Fig. 2B) results to be almost structureless and therefore do not allow any specific spectral recognition.

**References**

1. Hartmann N. I. B., Nolte T., Sørensen M., Jensen P. & Baun A. (2015). Aquatic ecotoxicity testing of nanoplastics. Lessons Learned From Nanoecotoxicology. DTU Environment. ASLO Aquatic Sciences Meeting 2015, Granada, Spain, 22/02/2015.
2. Chadha S., Ghiamati E., Manoharan R. & Nelson W. H. UV-Excited Raman and Resonance Raman Spectra of Synthetic Polymers. *Appl. spectros.* **46** (7), 1176-1181 (1992).

**Figures**


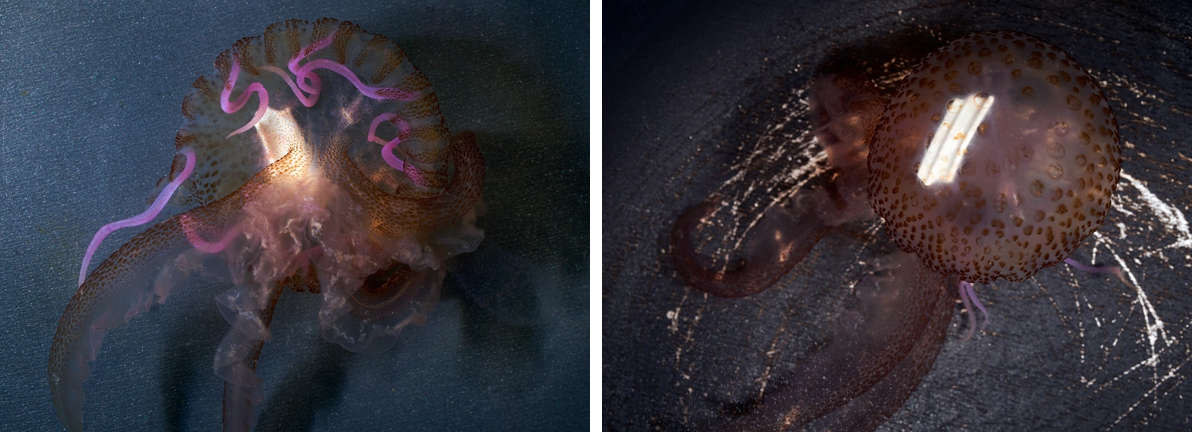


**Figure S1. Evidence of plastic ingestion in jellyfish.** A specimen of mauve stinger *P. noctiluca* seen from oral (left) and aboral (right) view, with a white polyethylene fragment (corresponding to the plastic piece shown in Figure 2B) inside the hood.


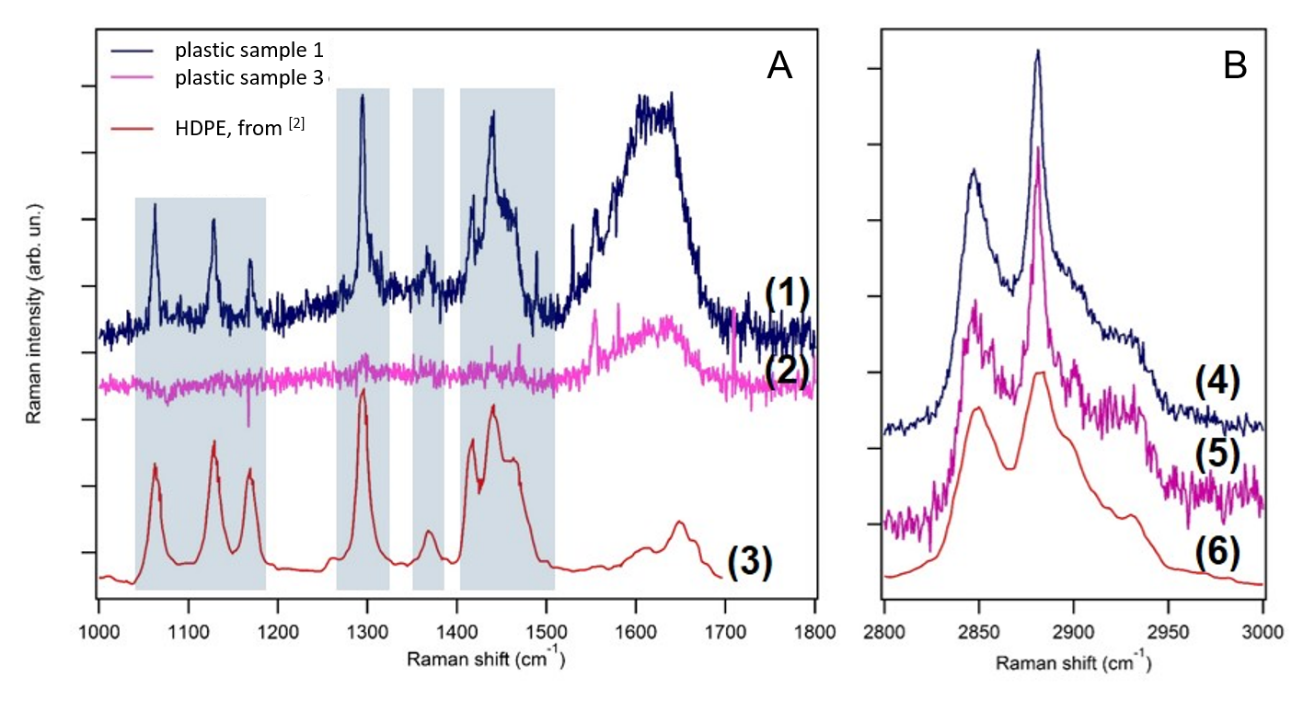


**Figure S2. UV-Raman spectra of the plastic debris found in *P. noctiluca* specimens.** Panel A: (1) showing spectral regions characteristic of HD-PE as major component (3), reported from ^[2]^, whereas (2) resulted less structured for an exact peak attribution. Panel B: region from 2900 cm^-1^, where similar spectra (4, 5, 6) were found for the three plastics.
